# Adaptive Synchronization of Fractional-Order Complex Chaotic system with Unknown Complex Parameters

**DOI:** 10.3390/e21020207

**Published:** 2019-02-21

**Authors:** Ruoxun Zhang, Yongli Liu, Shiping Yang

**Affiliations:** 1 College of Primary Education, Xingtai University, Xingtai 054001, China; 2 College of Physics Science and Information Engineering, Hebei Normal University, Shijiazhuang 050016, China

**Keywords:** synchronization, fractional-order, complex-variable chaotic system, unknown complex parameters

## Abstract

This paper investigates the problem of synchronization of fractional-order complex-variable chaotic systems (FOCCS) with unknown complex parameters. Based on the complex-variable inequality and stability theory for fractional-order complex-valued system, a new scheme is presented for adaptive synchronization of FOCCS with unknown complex parameters. The proposed scheme not only provides a new method to analyze fractional-order complex-valued system but also significantly reduces the complexity of computation and analysis. Theoretical proof and simulation results substantiate the effectiveness of the presented synchronization scheme.

## 1. Introduction

In the past 20 years, fractional-order chaotic systems have been extensively studied due to their wide applications in the fields of secure communication, control engineering, finance, physical and mathematical science, entropy, encryption and signal processing [1,2,3,4]. Meanwhile, synchronization of such systems has aroused tremendous attention of many researchers. Lots of excellent results were obtained and some methods of synchronization have been presented [5,6,7,8,9,10,11,12,13,14,15,16,17]. In various synchronization methods, the adaptive control approach is an effective method to realize the synchronization of uncertain systems.

The aforementioned works mainly investigated the fractional-order systems with real variables, not involving complex variables. Because complex variables that double the number of variables can generate complicated dynamical behaviors, enhance anti-attack ability and achieve higher transmission efficiency [18,19,20], many researchers have taken complex variables into the fractional-order systems and investigated dynamics behavior, stability, stabilization and synchronization of FOCCS in recent years. In [21,22,23], fractional-order complex-variable Chen system, T system and Lorenz system have been investigated, respectively. Recently, Zhang et al. [24] have investigated synchronization of fractional-order complex-valued delayed neural networks. Li et al. [25] presented adaptive synchronization scheme for fractional-order complex-valued neural networks with discrete and distributed delays. Sun et al. [26] proposed real combination synchronization of three fractional-order complex-variable chaotic systems, Yadav et al. [27] studied Dual function projective synchronization of fractional order complex chaotic systems, Nian et al. [28] realized synchronization of fractional-order complex chaotic system with parametric and external disturbances via sliding mode control method and Jiang et al. have studied complex modified projective synchronization (CMPS) for FOCCS in [29]. However, in these papers [24,25,26,27,28,29], the parameters of the FOCCS are exactly known in priori. In fact, in many practical engineering situations, most of system parameters cannot be accurately determined in advance and chaos synchronization will be destroyed with these uncertain factors. Hence, it is an important problem to realize synchronization of FOCCS with unknown complex parameters.

Inspired by the above discussions, the synchronization problem of FOCCS with unknown complex parameters was investigated in this paper. Using the inequality of the fractional derivative containing complex variable and the stability theory for fractional-order complex-valued system, we realized synchronization of such systems by constructing a suitable response system. It should be noted that we deal with the synchronization problem of fractional-order uncertain complex-variable system in complex-valued domain. That is to say, it is not necessary to separate the complex-variable system into its real and imaginary parts. This greatly reduces the complexity of computation and the difficulty of theoretical analysis.

Notation: ${\mathbb{C}}^{n}$ denotes complex *n*-dimensional space. For $z\in {\mathbb{C}}^{n}$, $z^{r}$, $z^{i}$, $\bar{z}$, $z^{T}$, $z^{H}$ and ||z|| are the real part, imaginary part, conjugate, transpose, conjugate transpose and *l*_2_-norm of z, respectively. For a matrix $A\in \mathbb{C}{\text{​}}^{n\;\times n}$, $A^{H}$ denotes its conjugate transpose.

## 2. Preliminaries

**Definition 1**  [30]**.** *The fractional integral of order α for a function f is defined as:*

(1) Iαf(t)=Dt−αt0f(t)=1Γ(α)∫t0t(t−τ)α−1f(τ)dτ

*where t ≥ t_0_ and α > 0.*

**Definition 2**  [30]**.** *Caputo’s fractional derivative of order* α *for a function* $f\in {\mathbb{R}}^{n}$ *is defined by:*

(2) Dtαt0Cf(t)=1Γ(n−α)∫t0tf(n)(τ)(t−τ)α−n+1dτ

*where t ≥ t_0_ and n is a positive integer such that n − < α < 1*.

**Lemma 1**  [31]**.** *Let* $x(t)\in {\mathbb{R}}^{n}$ *be a continuous and derivable vector function. Then, for any time instant t ≥ t_0_ and* α∈(0,1):

(3) 12Dtαt0C[xT(t)x(t)]≤xT(t)Dtαt0Cx(t)

**Corollary** **1.** *For a scalar derivable function* φ(t) *and a constants C, we have:*

(4) 12Dtαt0C(φ(t)−C)2≤(φ(t)−C)Dtαt0Cφ(t)

**Lemma 2**  [32] **.** *Let* $z\;\in {\mathbb{C}}^{n}$ *be a differentiable complex-valued vector. Then,* $\forall t\geq t_{0}$ *and* α∈(0, 1]*, the following inequality holds:*

(5) Dtαt0CzH(t)Pz(t)≤zH(t)PDtαt0Cz(t)+(Dtαt0Cz(t))HPz(t)

*where* $P\in {\mathbb{C}}^{n\times n}$ *is a constant positive definite Hermitian matrix*.

**Lemma** **3.** *For the fractional-order complex-variable systems:*

(6) Dtα0Cz(t)=h(z(t))

*where* 0<α<1 *,* $z(t)={\left(z_{1},z_{2},\cdots ,z_{n}\right)}^{T}\in {\mathbb{C}}^{n}$ *is the system complex state vector,* $h\in {\mathbb{C}}^{n}$ *is a continuous nonlinear function complex vector, which satisfies the globally Lipschitz continuity condition in the complex domain. Let* z(t)=0 *be an equilibrium point of system (1) and let* $V_{1}(t)=z^{H}(t)z(t)$ *and* $V_{2}(z(t))\geq 0$ *are continuously differentiable functions. If:*

(7) $$V(t)=V_{1}(t)+V_{2}(z(t))$$

*and:*

(8) Dtα0tV(t)≤−θV1(t)

*where* θ *is a positive constant. Then z(t) = 0 is asymptotically stable.*

**Proof.**  See the Appendix A. It was pointed out [33,34,35] that a similar theorem is obtained for the real systems.

**Remark** **1.** *Using Lemmas 2.2–2.3, one can directly analyze fractional order complex-variable system in the complex space.*

## 3. Main Results

We considered a kind of FOCCS described by:

(9) Dtα0Cz(t)=Az(t)+f(z(t))

where 0<α<1, $z(t)={\left(z_{1},z_{2},\cdots ,z_{n}\right)}^{T}\in {\mathbb{C}}^{n}$ is the system complex state vector, $f\in {\mathbb{C}}^{n}$ is a continuous nonlinear function vector, which satisfies the globally Lipschitz continuity condition in the complex domain and $A\in {\mathbb{C}}^{n\times n}$ is unknown (complex or real) parameter matrix. Furthermore, Equation (10) can be rewritten as:

(10) Dtα0Cz(t)=g(z(t))θ+f(z(t))

where $g:\;{\mathbb{C}}^{n}\to {\mathbb{C}}^{n\times m}$ is a complex function matrix, and $\theta ={\left(\theta_{1},\;\theta_{2},\;\cdots ,\;\theta_{m}\right)}^{T}$ is the system unknown complex parameter vector. For system (9) or (10), there are two propositions as follows.

**Proposition** **1.** *There exists a positive constant l_1_ such that the following inequality holds:*

(11) $${\left(z-w\right)}^{H}[g(z)-g(w)]\theta +{\left\{[g(z)-g(w)]\theta \right\}}^{H}(z-w)\leq l_{1}{\left(z-w\right)}^{H}(z-w)$$

**Proof.**  Given that g(z(t))θ=Az(t) results in:

$$\begin{array}{l} {\left(z-w\right)}^{H}[g(z)-g(w)]\theta +{\left\{[g(z)-g(w)]\theta \right\}}^{H}(z-w)\\ ={\left(z-w\right)}^{H}A\;(z-w)+{\left(z-w\right)}^{H}A^{H}(z-w)\\ ={\left(z-w\right)}^{H}(A+A^{H})(z-w) \end{array}$$

Since $A+A^{H}$ is Hermitian Matrix:

$$\lambda_{m}{\left(z-w\right)}^{H}(z-w)\leq {\left(z-w\right)}^{H}(A+A^{H})(z-w)\leq \lambda_{M}{\left(z-w\right)}^{H}(z-w)$$

where $\lambda_{m}$ and $\lambda_{M}$ are the minimum and maximum eigenvalue of $A+A^{H}$, respectively [36,37].

Let $l_{1}=\max(|\lambda_{m}|,|\lambda_{M}|)$, then, one has:

$${\left(z-w\right)}^{H}[g(z)-g(w)]\theta +{\left\{[g(z)-g(w)]\theta \right\}}^{H}(z-w)={\left(z-w\right)}^{H}(A+A^{H})(z-w)\leq l_{1}{\left(z-w\right)}^{H}(z-w)$$

**Proposition 2**  [38] **.** For the Lipschitz continuous function $f\in {\mathbb{C}}^{n}$, there exists a positive constant *l*_2_ such that the following inequality holds:

(12) $${\left(z-w\right)}^{H}[f(z)-f(w)]+{\left[f(z)-f(w)\right]}^{H}(z-w)\leq l_{2}{\left(z-w\right)}^{H}(z-w)$$

**Proof.**  For $f\in {\mathbb{C}}^{n}$, Lipschitz is continuous, then ||f(z)−f(w)|| ≤ L||z−w||, where L≥0 is a constant. It follows:

(z−w)H[f(z)−f(w)]+[f(z)−f(w)]H(z−w)=2Re{(z−w)H[f(z)−f(w)]}≤2|z−w|T|f(z)−f(w)| ≤  |z−w|T|z−w|+|f(z)−f(w)|T|f(z)−f(w)|=(z−w)H(z−w)+||f(z)−f(w)||2 ≤(z−w)H(z−w)+L2||z−w||2=(1+L2)(z−w)H(z−w)=l2(z−w)H(z−w)

where $l_{2}=L^{2}+1$, $|f(z)-f(w)|={\left(|f_{1}(z)-f_{1}(w)|,|f_{2}(z)-f_{2}(w)|,\cdots ,|f_{n}(z)-f_{n}(w)|\right)}^{T}$ and $|z-w|={\left(|z_{1}-w_{1}|,|z_{2}-w_{2}|,\cdots ,|z_{n}-w_{n}|\right)}^{T}$.

**Remark** **2.** *It is easy to check that many typical FOCCSs, such as the fractional-order complex-variable Chen system [21], T system [22] and Lorenz system [23] all satisfy Propositions 1 and 2.*

Choose system (11) as the master system, then the controlled response system is given by:

(13) Dtα0Cw(t)=g(w(t))θ^+f(w(t))+u(t)

where $w(t)={\left(w_{1},w_{2},\cdots ,w_{n}\right)}^{T}$ is the complex state vector, $\hat{\theta }\in {\mathbb{C}}^{m}$ represents the estimate vector of unknown vector θ, and $u(t)={\left(u_{1}(t),u_{2}(t),\cdots ,u_{n}(t)\right)}^{T}$ is controller to be determined.

**Theorem** **1.** *Asymptotically synchronization and parameter identification of systems (13) and (11) can be achieved under adaptive controller:*

(14) u(t)=−ke(t)

*and the complex update laws:*

(15) Dtα0Ck=σeH(t)e(t)

(16) Dtα0Ceθ=Dtα0Cθ^=−ηgH(w(t))e(t)

*where* e(t)=w(t)−z(t) *is the error vector,* $e_{\theta }=\hat{\theta }-\theta$ *is the parameter error, σ, η are two arbitrary positive constants.*

**Proof.**  From the error vector and systems (11) and (13), it yields:

Dtα0Ce(t)=g(w(t))θ^+f(w(t))−g(z(t))θ+f(z(t))+u(t)=g(w(t))eθ+[g(w(t))−g(z(t))]θ+f(w(t))−f(z(t))+u(t)

Let us present the Lyapunov function:

(17) $$V(t,e(t))=e^{H}(t)e(t)+\frac{1}{\sigma }{\left(k-k^{*}\right)}^{2}+\frac{1}{\eta }e_{\theta }^{H}(t)e_{\theta }(t)$$

where $k^{*}$ is to be determined.

Using Lemma 2.1, Corollary 2.1 and Lemma 2.2, we have:

Dtα0CV(t,e(t))=Dtα0C[eH(t)e(t)+1σ(k−k*)2+1ηeθH(t)eθ(t)]≤eH(t)Dtα0Ce(t)+[Dtα0Ce(t)]He(t)+2σ(k−k*)Dtα0Ck+1ηeθH(t)Dtα0Ceθ(t)+1η[Dtα0Ceθ(t)]Heθ(t)≤eH(t){g(w(t))eθ+[g(w(t))−g(z(t))]θ+f(w(t))−f(z(t))−ke(t)}+{g(w(t))eθ +[g(w(t))−g(z(t))]θ +f(w(t))−f(z(t))−ke(t)}He+2σ(k−k*)Dtα0Ck +1ηeθH(t)Dtα0Ceθ(t)+1η[Dtα0Ceθ(t)]Heθ(t)≤eH(t){g(w(t))eθ+[g(w(t))−g(z(t))]θ+f(w(t))−f(z(t))−ke(t)}+{g(w(t))eθ +[g(w(t))−g(z(t))]θ +f(w(t))−f(z(t))−ke(t)}He+2σ(k−k*)Dtα0Ck +1ηeθH(t)Dtα0Ceθ(t)+1η[Dtα0Ceθ(t)]Heθ(t) ≤eH(t){g(w(t))eθ+[g(w(t))−g(z(t))]θ+f(w(t))−f(z(t))−ke(t)}+{g(w(t))eθ  +[g(w(t))−g(z(t))]θ +f(w(t))−f(z(t))−ke(t)}He+2σ(k−k*)Dtα0Ck  +1ηeθH(t)Dtα0Ceθ(t)+1η[Dtα0Ceθ(t)]Heθ(t)

Substitute Equations (15) and (16) into the inequality above, we further have:

Dtα0CV(t,e(t)) ≤eH(t){g(w(t))eθ+[g(w(t))−g(z(t))]θ+f(w(t))−f(z(t))−ke(t)}  +{g(w(t))eθ+[g(w(t))−g(z(t))]θ+f(w(t))−f(z(t))−ke(t)}He  +2(k−k*)eH(t)e(t)−eθH(t)gH(w(t))e(t)−[gH(w(t))e(t)]Heθ(t)   ≤eH(t)[g(w(t)−g(z(t))]θ+{[g(w(t)−g(z(t))]θ}He(t)   +eH(t)[f(w(t)−f(z(t))]+[f(w(t)−f(z(t))]He(t)−2k*eH(t)e(t)

From Propositions 1 and 2, we can obtain:

$$e^{H}(t)[g(w(t))-g(z(t)]\theta +\{\left[g(w(t)\right){-g(z(t)]\theta \}}^{H}e(t)\leq l_{1}e^{H}(t)e(t)$$

and:

$$e^{H}(t)[f(w(t))-f(z(t)]+[f(w(t))-f{\left(z(t)\right]}^{H}e(t)\leq l_{2}e^{H}(t)e(t)$$

then, one has:

Dtα0CV(t,e(t))≤eH(t)[l1+(l2−2k*)I]e(t)

Let $2k^{*}=l_{1}+l_{2}+1$, then:

(18) Dtα0CV(t,e(t)) ≤−eH(t)e(t)

According to Lemma 2.3, one has $\underset{t\to \infty }{\lim}e^{H}(t)e(t)=0,$ which implies $\underset{t\to \infty }{\lim}e(t)=0$, which shows that the systems (11) and (13) can obtain asymptotically synchronization. Meanwhile, according to Remark 1 of Theorem 1 in [39], parameter identification is achieved.

**Remark** **3.** *In previous work [24,25,26,27,28,29], the common method to analyze fractional complex-valued systems is to separate into two real-valued systems according to their real and imaginary parts, and then the criteria on synchronization were obtained by investigating these real-valued systems. However, there are two problems with this approach. One is that the dimension of the real-valued system is twice that of the original complex-valued system, which adds the complexity of computation and analysis. The other is that this method requires that complex-valued functions be explicitly separated into real and imaginary parts. However, this separation is not always expressible in an analytical form. Unlike from previous works, in our proposed method, the entire analysis process is performed in the complex-valued domain, and the complex function theory is used to derive synchronization conditions without separating the original complex-valued chaotic system into two real-valued systems, which reduces the complexity of analysis and computation. Moreover, the proposed method can be applied to other complex-valued systems, such as complex networks with fractional-order complex-variable dynamics and fractional-order complex-valued neural network systems.*

**Remark** **4.** *If the system parameters are known, the update law will be reduced to (15) only.*

## 4. Numerical Simulations

In this section, in order to show the effectiveness of the proposed scheme in preceding section, numerical example on fractional-order complex chaotic system will be provided. When numerically solving such systems, we first adopt the predictor–corrector method [40] by MATLAB. Lyapunov exponents of the systems are calculated by adopting the Wolf et al. algorithm [41] with some changes.

**Figure 1:**
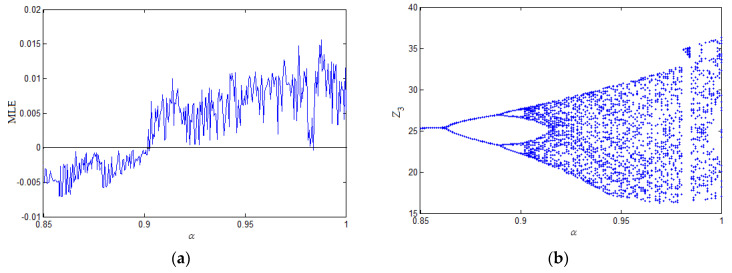
Dynamic behaviors of the fractional-order complex Lorenz-like System with commensurate order α (a=10+i, b=3, c=16+0.3i). (**a**) maximal Lyapunov exponent; (**b**) bifurcation diagram.

**Figure 2:**
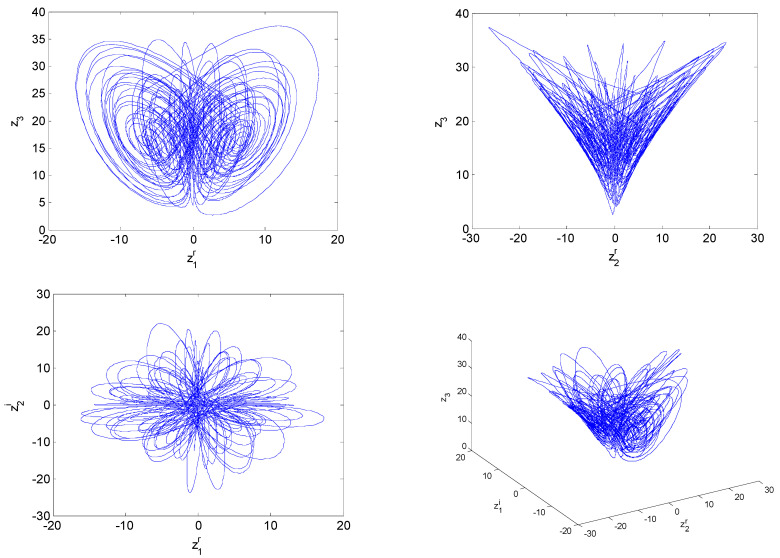
Chaotic attractors of fractional-order complex Lorenz-like system with a=10+i, b=3, c=16+0.3i and commensurate order α=0.95.

Consider the Lorenz-like fractional-order complex chaotic system with commensurate order:

(19) (Dtα0Cz1Dtα0Cz2Dtα0Cz3)=(a(z2+z1)−cz2−z1z3z¯1z1−bz3)=(z2+z1000−z2000−z3)(acb)+(0−z1z3z¯1z1)

where $z_{1},\;z_{2},\;z_{3}$ are the complex state variables and *a*, *b*, *c* are system parameters; let a=10+i, b=3, c=16+0.3i. The maximum Lyapunov exponent (MLE) spectrum is depicted in Figure 1a, and the bifurcation diagram is presented in Figure 1b. Figure 1a,b shows that system (19) is chaotic with fractional order α∈[0.91,0.98]∪[0.985,1] and parameters a=10+i, b=3, c=16+0.3i. When the fractional-order α=0.95, the attractor trajectories are illustrated in Figure 2.

Recently, ref. [42] described how to perform a successful simulation and optimization, and how to synthesize the mathematical models using CMOS technology. The application of metaheuristics to optimize MLE by varying the parameters of the oscillators was discussed.

**Figure 3:**
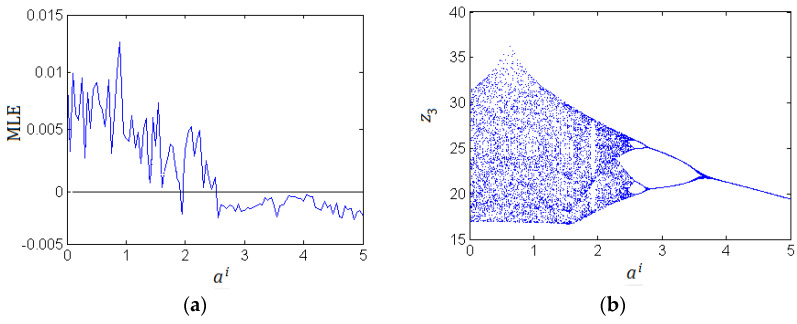
Dynamic behaviors of the fractional-order complex Lorenz-like System with commensurate order 0.95 ($a^{r}=10,\;b=3,\;c=16+0.3i$). (**a**) maximal Lyapunov exponent; (**b**) bifurcation diagram.

**Figure 4:**
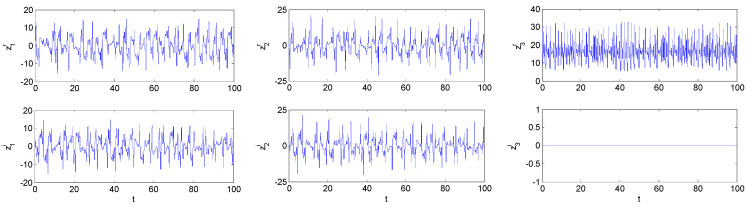
The state trajectories of fractional-order complex Lorenz-like system with a=10+i, b=3, c=16+0.3i and commensurate order α=0.95.

Field-programmable gate array (FPGA)-based implementation of chaotic oscillators has demonstrated its usefulness in the development of engineering applications in a wide variety of fields, such as: random number generators, robotics and chaotic secure communication systems, signal processing. Very recently, Pano-Azucena et al. [43] implemented the chaotic system using a field-programmable gate array (FPGA) based on trigonometric polynomials. Reference [44] detailed the FPGA-based implementation of all the fractional order chaotic oscillators applying Grünwald-Letnikov(G-L) method. Their work proved experimentally that applying G-L method with 256 elements of memory; it can observe different families of Fractional-order chaotic attractors having working frequencies between 77.59 MHz and 84.9 MHz. This is very beneficial to the development of fractional-order chaos in engineering applications. For the FPGA-based implementation of FOCCS, the FOCCS was first separated into two real-variable systems according to their real and imaginary parts, and then these real-variable systems can be implemented using FPGA by the method proposed by. In order to find much better behavior and characteristics of the FOCCS in the complex domain, we used the G-L method to numerically solve the system (19) again. The MLE spectrum with varying parameter *a^i^* (the imaginary of *a*) is depicted in Figure 3a, the bifurcation diagram is presented in Figure 3b, the state trajectories are illustrated in Figure 4.

Taking the system (19) as master system, and assuming the parameters *a*, *b* and *c* are unknown, the response system is given as follows:

(20) (Dtα0Cw1Dtα0Cw2Dtα0Cw3)=(w2+w1000−w2000−w3)(a^c^b^)+(0−w1w3w¯1w1)+(u1u2u3)

where $\hat{a},\;\hat{b},\;\hat{c}$ are parameter estimations. $u_{1},\;u_{2},\;u_{3}$ are the controller.

According to Theorem 1, the controllers and the update rules are selected as:

(21) u1=−ke1, u2=−ke2, u3=−ke3Dtα0Ck=σeHe=σ(e¯1e1+e¯2e2+e¯3e3), (σ>0)

(22) (Dtα0Ca^Dtα0Cc^Dtα0Cb^)=(w1+w2000−w2000−w3)H(e1e2e3)=(−(w1+w2)e1w¯2e2w¯3e3)

**Figure 5:**
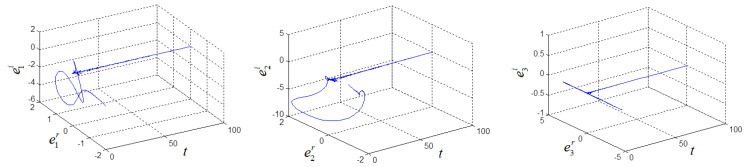
Synchronization errors *e*_1_, *e*_2_, *e*_3_ of fractional-order complex Lorenz-like chaotic system.

**Figure 6:**
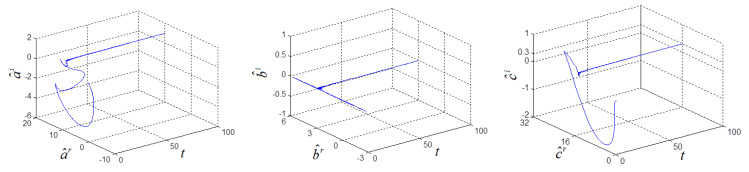
Estimated complex parameters of fractional-order complex Lorenz-like chaotic system.

In the simulation, let α=0.95, (*a*, *b*, *c*) = (30 + *i*, 3, 26 + 0.6*i*), the initial conditions z(0)= ${\left(1+i,\;-2-i,\;6\right)}^{T},\;w(0)={\left(-1+i,\;-3+i,\;10\right)}^{T},$ $\left(\hat{a}(0),\;\hat{b}(0),\;\hat{c}(0))=(20,2,20\right)$, k(0)=0 and σ=6. Two systems can achieve synchronization and the parameters are identified, as shown in Figure 5 and Figure 6.

## 5. Conclusions

We studied the adaptive synchronization of FOCCS with unknown complex parameters, and proposed a method for analyzing FOCCS without separating system into real and imaginary parts. By this method, the constructed response system can be asymptotically synchronized to an uncertain drive system with a desired complex scaling diagonal matrix. The proposed synchronization scheme retains the complex nature of fractional-order complex chaotic system. It not only provides a new method of analyzing FOCCS, but also significantly decreases the complexity of computation and analysis. We hope that the work performed will be helpful to further research of nonlinear fractional order complex-variable systems.

## Appendix A

**Proof of Lemma 3.** By α-integrating (8), we have:

(A1) $$V(t)-V(0)\leq -\theta I^{\alpha }V_{1}(t)=-\theta I^{\alpha }z^{H}(t)z(t)\leq 0$$

Thus, V(t)≤V(0), t≥0. From (6), we can obtain that $V_{1}(t)=z^{H}(t)z(t)$ is bounded. Furthermore, since *h*(*z*(*t*)) satisfies the globally Lipschitz continuity condition, from (6), one has, ||Dtα0Cz(t)|| =||h(z(t))|| $\leq l||z^{H}(t)z(t))||$, i.e., [∑i=1n(Dtα0Czi(t)¯ Dtα0Czi(t) )]1/2≤l [∑i=1n (zi(t)¯ zi(t))]1/2, where *l* is positive constant. Given that $V_{1}(t)=z^{H}(t)z(t)$ is bounded, we have $\left|z_{i}\right|$ is bounded, and then |Dtα0Czi(t)| is bounded. Thus, there exists a constant *M* > 0, such that |Dtα0Czi(t)|≤M. For $0\leq t_{1}<t_{2}$ and any ε>0, if $\left|t_{2}-t_{1}|<\delta (\varepsilon \right)$ $={\left[\frac{\varepsilon \text{​}\Gamma (\alpha +1)}{2M}\right]}^{\frac{1}{\alpha }}$, one can get:

|zi(t1)−zi(t2)|=|Dt−α0Dtα0Czi(t1)+zi(0)−[Dt−α0Dtα0Czi(t2)+zi(0)]|=|Dt−α0Dtα0Czi(t1)−Dt−α0Dtα0Czi(t2)|=1Γ(α)|∫0t1[(t1−τ)α−1−(t2−τ)α−1]Dτα0Czi(τ)dτ−∫t1t2(t2−τ)α−1Dτα0Czi(τ)dτ|≤MΓ(α){∫0t1[(t1−τ)α−1−(t2−τ)α−1]dτ+∫t1t2(t2−τ)α−1dτ}=MΓ(α+1)[(t1α−t2α)+2(t2−t1)α]≤2MΓ(α+1)(t2−t1)α≤ε

Hence, $z_{i}(t)$ is uniformly continuous. Therefore, $V_{1}(t)=z^{H}(t)z(t)$ is uniformly continuous.

Next, we adopt contradiction to prove $\underset{t\to \infty }{\lim}V_{1}(t)=\underset{t\to \infty }{\lim}z^{H}(t)z(t)=0$ using the idea of Theorem 1 in Reference [45].

Suppose that $V_{1}(t)\neq 0$ as t→∞. Then there exists a monotone increasing sequence ${\left(t_{k}\right)}_{k\in N^{\;+}}$ ($t_{k}\to \infty$ as k→∞) and a positive constant ε > 0 such that $V_{1}(t_{k})>\varepsilon$. Since the uniform continuity of $V_{1}(t)$, for the given ε, $\exists \delta >0\;\;(\delta \leq \inf_{j\in N^{+}}\{t_{j+1}-t_{j}\},$ which implies that the intervals $\left[t_{k},t_{k}+\delta \right]$ are nonoverlapping) such that $|V_{1}(t_{k})-V_{1}(t)|\leq \varepsilon /2$. Then, for any $t\in [t_{k},t_{k}+\delta ]$ it follows that $V_{1}(t)=\;V_{1}(t_{k})-V_{1}(t_{k})+V_{1}(t)$ $\geq \;|V_{1}(t_{k})|-|V_{1}(t_{k})-V_{1}(t)|\;>\varepsilon /2$. Thus, for any *k* = 1, 2, 3, …, from (A1), we have:

$$\begin{array}{cc} V(t_{k}+\delta )-V(0) & \leq \frac{-\theta }{\Gamma (\alpha )}\int_{0}^{t_{k}+\delta }\frac{V_{1}(\tau )}{{\left(t_{k}+\delta -\tau \right)}^{1-\alpha }}d\tau \;\\ & =\frac{-\theta }{\Gamma (\alpha )}[\int_{0}^{t_{1}}+\;\int_{t_{1}}^{t_{1}+\delta }+\;\int_{t_{1}+\delta }^{t_{2}}+\;\int_{t_{2}}^{t_{2}+\delta }+\int_{t_{2}+\delta }^{t_{3}}+\;\int_{t_{3}}^{t_{3}+\delta }+\;\cdots \;+\int_{t_{k}}^{t_{k}+\delta }]\;\frac{V_{1}(\tau )}{{\left(t_{k}+\delta -\tau \right)}^{1-\alpha }}d\tau \\ & \;\leq \frac{-\theta }{\Gamma (\alpha )}[\int_{t_{1}}^{t_{1}+\delta }+\int_{t_{2}}^{t_{2}+\delta }+\;\int_{t_{3}}^{t_{3}+\delta }+\;\cdots +\int_{t_{k}}^{t_{k}+\delta }]\;\frac{V_{1}(\tau )}{{\left(t_{k}+\delta -\tau \right)}^{1-\alpha }}d\tau \\ & \leq \frac{-\theta \varepsilon }{2\Gamma (\alpha )}[\int_{t_{1}}^{t_{1}+\delta }+\int_{t_{2}}^{t_{2}+\delta }+\;\int_{t_{3}}^{t_{3}+\delta }+\;\cdots +\int_{t_{k}}^{t_{k}+\delta }]\;{\left(t_{k}+\delta -\tau \right)}^{\alpha -1}d\tau \\ & =\frac{-\theta \varepsilon }{2\Gamma (\alpha )}\sum_{j=1}^{k}\int_{t_{j}}^{t_{j}+\delta }\;{\left(t_{k}+\delta -\tau \right)}^{\alpha -1}\;d\tau \end{array}$$

Given that ${\left(t_{k}+\delta -\tau \right)}^{\alpha -1}\geq {\left(t_{k}+\delta -t_{j}\right)}^{\alpha -1}$ for all $\tau \in [t_{j},\;t_{j}+\delta ]$ results in:

$$\begin{array}{cc} V(t_{k}+\delta )-V(0) & \leq \frac{-\theta \varepsilon }{2\Gamma (\alpha )}\sum_{j=1}^{k}\int_{t_{j}}^{t_{j}+\delta }\;{\left(t_{k}+\delta -\tau \right)}^{\alpha -1}\;d\tau \\ & \leq \frac{-\theta \varepsilon }{2\Gamma (\alpha )}\sum_{j=1}^{k}\int_{t_{j}}^{t_{j}+\delta }\;{\left(t_{k}+\delta -t_{j}\right)}^{\alpha -1}\;d\tau \\ & =\frac{-\theta \varepsilon \delta }{2\Gamma (\alpha )}\sum_{j=1}^{k}{\left(t_{k}+\delta -t_{j}\right)}^{\alpha -1}\\ & \leq \frac{-\theta \varepsilon \delta }{2\Gamma (\alpha )}\sum_{j=1}^{k}{\left(t_{k}+\delta -t_{1}\right)}^{\alpha -1}\\ & =\frac{-\theta \varepsilon \delta }{2\Gamma (\alpha )}\frac{k}{{\left(t_{k}+\delta -t_{1}\right)}^{1-\alpha }}\\ & \leq \frac{-\theta \varepsilon \delta }{2\Gamma (\alpha )}\;\frac{k}{{\left(kd\right)}^{1-\alpha }}\;=\frac{-\theta \varepsilon \delta }{2\Gamma (\alpha )}\;\frac{k^{\alpha }}{{\left(d\right)}^{1-\alpha }} \end{array}$$

where $d=\sup_{j\in N,\;2\;\leq j\;\leq k}\{t_{j}-t_{j-1}\}$ (since $V_{1}(t)$ is a uniformly continuous function and assumed $V_{1}(t)\neq 0$, as t→∞, *d* is bounded). Obviously, $V(t_{k}+\delta )-V(0)\leq \frac{-\theta \varepsilon \delta }{2\Gamma (\alpha )}\;\frac{k^{\alpha }}{{\left(d\right)}^{1-\alpha }}\to -\infty$ as k→∞, which contradict with V(t)≥0. Therefore, $\underset{t\to \infty }{\lim}V_{1}(t)=\underset{t\to \infty }{\lim}z^{H}(t)z(t)=0$. i.e., *z*(*t*) = 0 is asymptotically stable. This completes the proof for Lemma 3.
